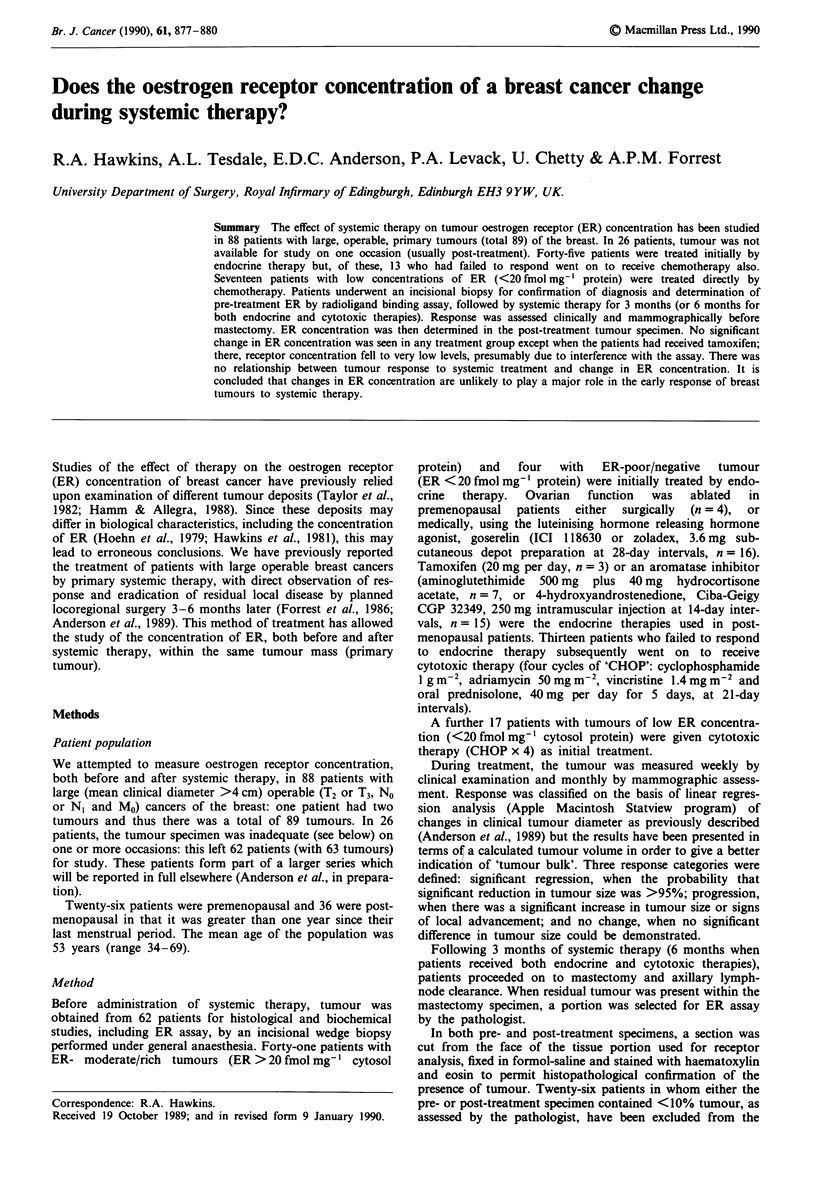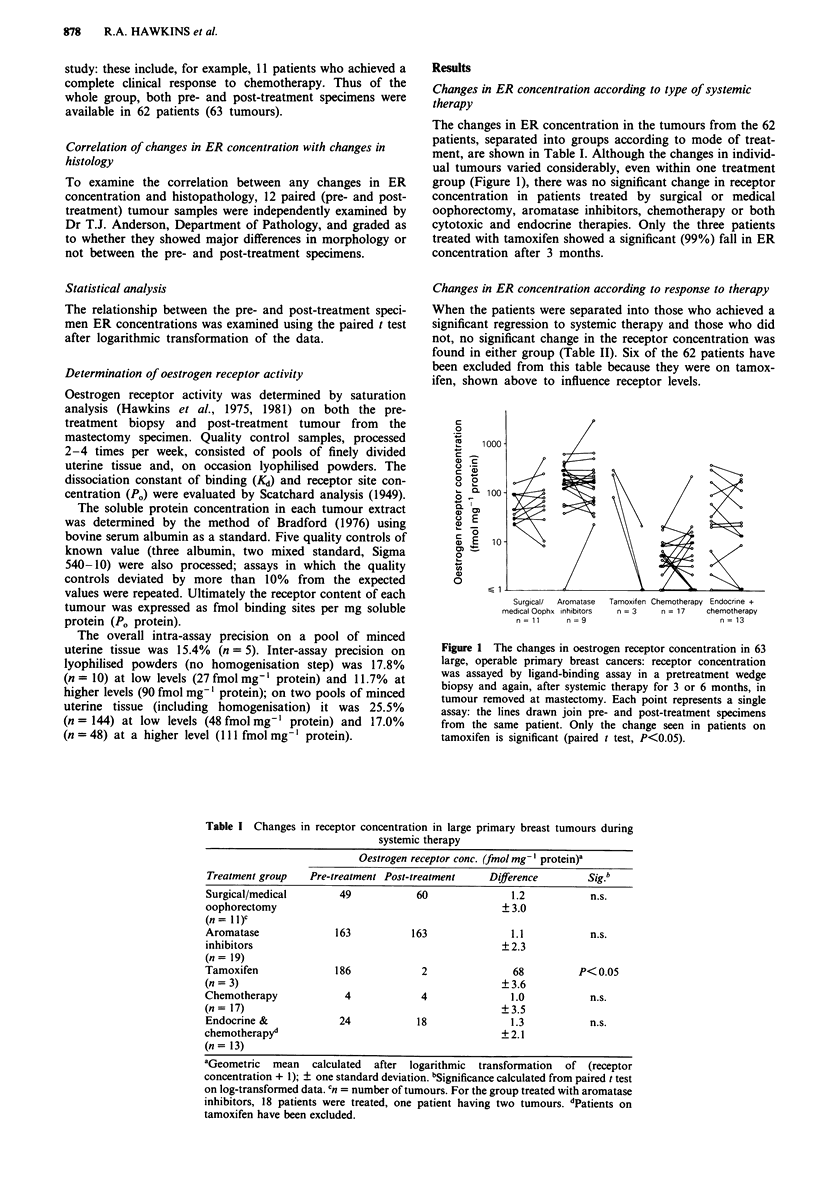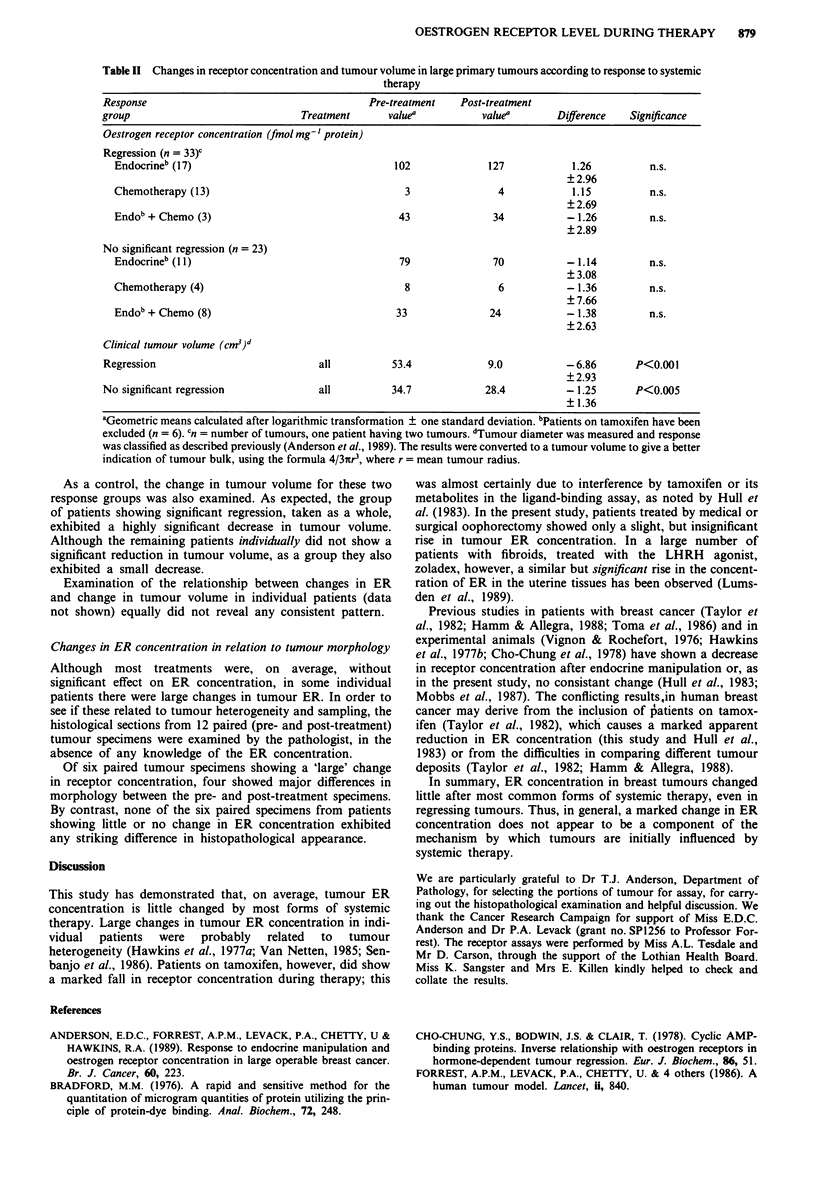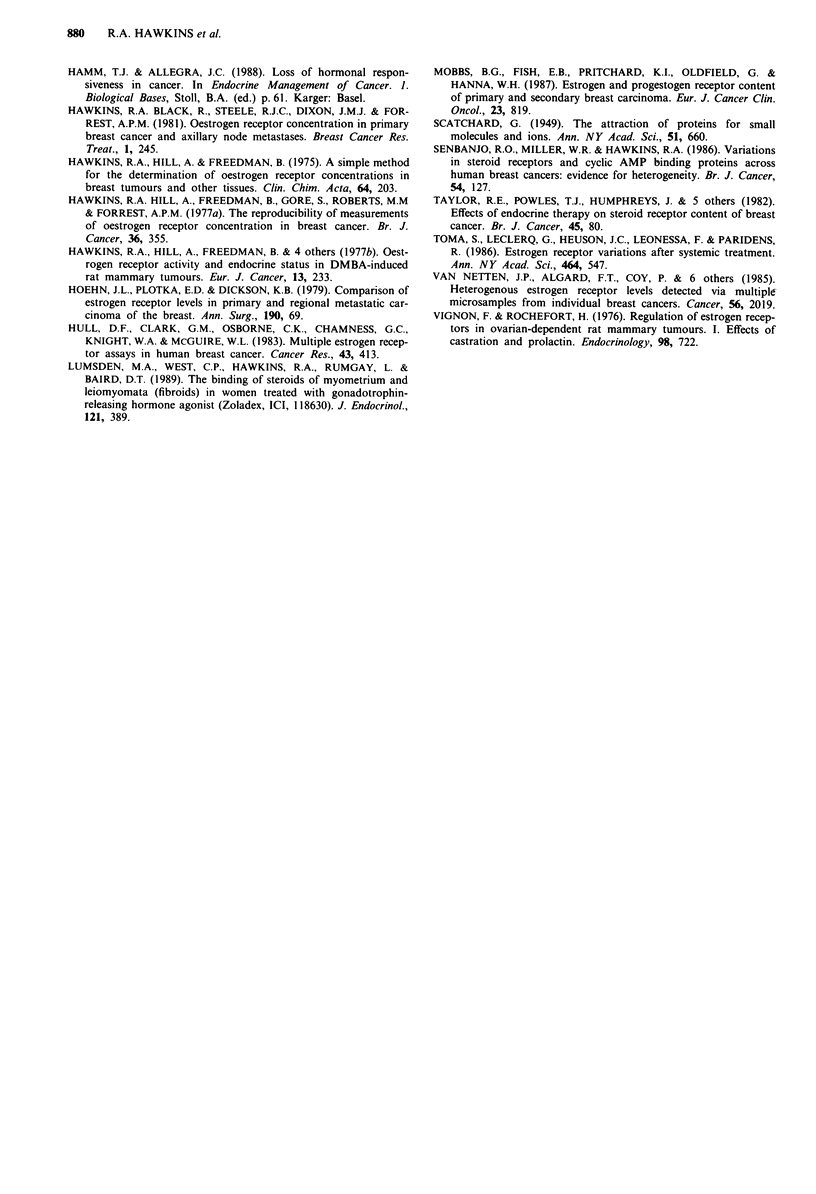# Does the oestrogen receptor concentration of a breast cancer change during systemic therapy?

**DOI:** 10.1038/bjc.1990.196

**Published:** 1990-06

**Authors:** R. A. Hawkins, A. L. Tesdale, E. D. Anderson, P. A. Levack, U. Chetty, A. P. Forrest

**Affiliations:** University Department of Surgery, Royal Infirmary of Edingburgh, UK.

## Abstract

The effect of systemic therapy on tumour oestrogen receptor (ER) concentration has been studied in 88 patients with large, operable, primary tumours (total 89) of the breast. In 26 patients, tumour was not available for study on one occasion (usually post-treatment). Forty-five patients were treated initially by endocrine therapy but, of these, 13 who had failed to respond went on to receive chemotherapy also. Seventeen patients with low concentrations of ER (less than 20 fmol mg-1 protein) were treated directly by chemotherapy. Patients underwent an incisional biopsy for confirmation of diagnosis and determination of pre-treatment ER by radioligand binding assay, followed by systemic therapy for 3 months (or 6 months for both endocrine and cytotoxic therapies). Response was assessed clinically and mammographically before mastectomy. ER concentration was then determined in the post-treatment tumour specimen. No significant change in ER concentration was seen in any treatment group except when the patients had received tamoxifen; there, receptor concentration fell to very low levels, presumably due to interference with the assay. There was no relationship between tumour response to systemic treatment and change in ER concentration. It is concluded that changes in ER concentration are unlikely to play a major role in the early response of breast tumours to systemic therapy.


 © Macmillan Press Ltd., 1990

### Does the oestrogen receptor concentration of a breast cancer change during systemic therapy?

R.A. Hawkins, A.L. Tesdale, E.D.C. Anderson, P.A. Levack, U. Chetty & A.P.M. Forrest

*University Department of Surgery, Royal Infirmary of Edingburgh, Edinburgh EH3 9YW, UK.*

**Summary** The effect of systemic therapy on tumour oestrogen receptor (ER) concentration has been studied in 88 patients with large, operable, primary tumours (total 89) of the breast. In 26 patients, tumour was not available for study on one occasion (usually post-treatment). Forty-five patients were treated initially by endocrine therapy but, of these, 13 who had failed to respond went on to receive chemotherapy also. Seventeen patients with low concentrations of ER (<20 fmol mg$^{-1}$ protein) were treated directly by chemotherapy. Patients underwent an incisional biopsy for confirmation of diagnosis and determination of pre-treatment ER by radioligand binding assay, followed by systemic therapy for 3 months (or 6 months for both endocrine and cytotoxic therapies). Response was assessed clinically and mammographically before mastectomy. ER concentration was then determined in the post-treatment tumour specimen. No significant change in ER concentration was seen in any treatment group except when the patients had received tamoxifen; there, receptor concentration fell to very low levels, presumably due to interference with the assay. There was no relationship between tumour response to systemic treatment and change in ER concentration. It is concluded that changes in ER concentration are unlikely to play a major role in the early response of breast tumours to systemic therapy.

Studies of the effect of therapy on the oestrogen receptor (ER) concentration of breast cancer have previously relied upon examination of different tumour deposits (Taylor *et al.*, 1982; Hamm & Allegra, 1988). Since these deposits may differ in biological characteristics, including the concentration of ER (Hoehn *et al.*, 1979; Hawkins *et al.*, 1981), this may lead to erroneous conclusions. We have previously reported the treatment of patients with large operable breast cancers by primary systemic therapy, with direct observation of response and eradication of residual local disease by planned locoregional surgery 3-6 months later (Forrest *et al.*, 1986; Anderson *et al.*, 1989). This method of treatment has allowed the study of the concentration of ER, both before and after systemic therapy, within the same tumour mass (primary tumour).

### Methods

### *Patient population*

We attempted to measure oestrogen receptor concentration, both before and after systemic therapy, in 88 patients with large (mean clinical diameter >4 cm) operable ($T_2$ or $T_3$, $N_0$ or $N_1$ and $M_0$) cancers of the breast: one patient had two tumours and thus there was a total of 89 tumours. In 26 patients, the tumour specimen was inadequate (see below) on one or more occasions: this left 62 patients (with 63 tumours) for study. These patients form part of a larger series which will be reported in full elsewhere (Anderson *et al.*, in preparation).

Twenty-six patients were premenopausal and 36 were postmenopausal in that it was greater than one year since their last menstrual period. The mean age of the population was 53 years (range 34-69).

### *Method*

Before administration of systemic therapy, tumour was obtained from 62 patients for histological and biochemical studies, including ER assay, by an incisional wedge biopsy performed under general anaesthesia. Forty-one patients with ER- moderate/rich tumours (ER > 20 fmol mg$^{-1}$ cytosol protein) and four with ER-poor/negative tumour (ER < 20 fmol mg$^{-1}$ protein) were initially treated by endocrine therapy. Ovarian function was ablated in premenopausal patients either surgically ($n = 4$), or medically, using the luteinising hormone releasing hormone agonist, goserelin (ICI 118630 or zoladex, 3.6 mg subcutaneous depot preparation at 28-day intervals, $n = 16$). Tamoxifen (20 mg per day, $n = 3$) or an aromatase inhibitor (aminoglutethimide 500 mg plus 40 mg hydrocortisone acetate, $n = 7$, or 4-hydroxyandrostenedione, Ciba-Geigy CGP 32349, 250 mg intramuscular injection at 14-day intervals, $n = 15$) were the endocrine therapies used in postmenopausal patients. Thirteen patients who failed to respond to endocrine therapy subsequently went on to receive cytotoxic therapy (four cycles of 'CHOP': cyclophosphamide 1 g m$^{-2}$, adriamycin 50 mg m$^{-2}$, vincristine 1.4 mg m$^{-2}$ and oral prednisolone, 40 mg per day for 5 days, at 21-day intervals).

A further 17 patients with tumours of low ER concentration (<20 fmol mg$^{-1}$ cytosol protein) were given cytotoxic therapy (CHOP × 4) as initial treatment.

During treatment, the tumour was measured weekly by clinical examination and monthly by mammographic assessment. Response was classified on the basis of linear regression analysis (Apple Macintosh Statview program) of changes in clinical tumour diameter as previously described (Anderson *et al.*, 1989) but the results have been presented in terms of a calculated tumour volume in order to give a better indication of 'tumour bulk'. Three response categories were defined: significant regression, when the probability that significant reduction in tumour size was >95%; progression, when there was a significant increase in tumour size or signs of local advancement; and no change, when no significant difference in tumour size could be demonstrated.

Following 3 months of systemic therapy (6 months when patients received both endocrine and cytotoxic therapies), patients proceeded on to mastectomy and axillary lymph-node clearance. When residual tumour was present within the mastectomy specimen, a portion was selected for ER assay by the pathologist.

In both pre- and post-treatment specimens, a section was cut from the face of the tissue portion used for receptor analysis, fixed in formol-saline and stained with haematoxylin and eosin to permit histopathological confirmation of the presence of tumour. Twenty-six patients in whom either the pre- or post-treatment specimen contained <10% tumour, as assessed by the pathologist, have been excluded from the

Correspondence: R.A. Hawkins.
Received 19 October 1989; and in revised form 9 January 1990.

study: these include, for example, 11 patients who achieved a complete clinical response to chemotherapy. Thus of the whole group, both pre- and post-treatment specimens were available in 62 patients (63 tumours).

*Correlation of changes in ER concentration with changes in histology*

To examine the correlation between any changes in ER concentration and histopathology, 12 paired (pre- and post-treatment) tumour samples were independently examined by Dr T.J. Anderson, Department of Pathology, and graded as to whether they showed major differences in morphology or not between the pre- and post-treatment specimens.

*Statistical analysis*

The relationship between the pre- and post-treatment specimen ER concentrations was examined using the paired *t* test after logarithmic transformation of the data.

*Determination of oestrogen receptor activity*

Oestrogen receptor activity was determined by saturation analysis (Hawkins *et al.*, 1975, 1981) on both the pre-treatment biopsy and post-treatment tumour from the mastectomy specimen. Quality control samples, processed 2–4 times per week, consisted of pools of finely divided uterine tissue and, on occasion lyophilised powders. The dissociation constant of binding ($K_d$) and receptor site concentration ($P_o$) were evaluated by Scatchard analysis (1949).

The soluble protein concentration in each tumour extract was determined by the method of Bradford (1976) using bovine serum albumin as a standard. Five quality controls of known value (three albumin, two mixed standard, Sigma 540–10) were also processed; assays in which the quality controls deviated by more than 10% from the expected values were repeated. Ultimately the receptor content of each tumour was expressed as fmol binding sites per mg soluble protein ($P_o$ protein).

The overall intra-assay precision on a pool of minced uterine tissue was 15.4% ($n = 5$). Inter-assay precision on lyophilised powders (no homogenisation step) was 17.8% ($n = 10$) at low levels (27 fmol mg$^{-1}$ protein) and 11.7% at higher levels (90 fmol mg$^{-1}$ protein); on two pools of minced uterine tissue (including homogenisation) it was 25.5% ($n = 144$) at low levels (48 fmol mg$^{-1}$ protein) and 17.0% ($n = 48$) at a higher level (111 fmol mg$^{-1}$ protein).

### Results

*Changes in ER concentration according to type of systemic therapy*

The changes in ER concentration in the tumours from the 62 patients, separated into groups according to mode of treatment, are shown in Table I. Although the changes in individual tumours varied considerably, even within one treatment group (Figure 1), there was no significant change in receptor concentration in patients treated by surgical or medical oophorectomy, aromatase inhibitors, chemotherapy or both cytotoxic and endocrine therapies. Only the three patients treated with tamoxifen showed a significant (99%) fall in ER concentration after 3 months.

*Changes in ER concentration according to response to therapy*

When the patients were separated into those who achieved a significant regression to systemic therapy and those who did not, no significant change in the receptor concentration was found in either group (Table II). Six of the 62 patients have been excluded from this table because they were on tamoxifen, shown above to influence receptor levels.

**Figure 1** The changes in oestrogen receptor concentration in 63 large, operable primary breast cancers: receptor concentration was assayed by ligand-binding assay in a pretreatment wedge biopsy and again, after systemic therapy for 3 or 6 months, in tumour removed at mastectomy. Each point represents a single assay: the lines drawn join pre- and post-treatment specimens from the same patient. Only the change seen in patients on tamoxifen is significant (paired *t* test, $P<0.05$).

**Table I** Changes in receptor concentration in large primary breast tumours during systemic therapy

| | *Oestrogen receptor conc. (fmol mg$^{-1}$ protein)*[a] | | | |
|---|---|---|---|---|
| *Treatment group* | *Pre-treatment* | *Post-treatment* | *Difference* | *Sig.*[b] |
| Surgical/medical oophorectomy ($n = 11$)[c] | 49 | 60 | 1.2 ±3.0 | n.s. |
| Aromatase inhibitors ($n = 19$) | 163 | 163 | 1.1 ±2.3 | n.s. |
| Tamoxifen ($n = 3$) | 186 | 2 | 68 ±3.6 | $P<0.05$ |
| Chemotherapy ($n = 17$) | 4 | 4 | 1.0 ±3.5 | n.s. |
| Endocrine & chemotherapy[d] ($n = 13$) | 24 | 18 | 1.3 ±2.1 | n.s. |

[a]Geometric mean calculated after logarithmic transformation of (receptor concentration + 1); ± one standard deviation. [b]Significance calculated from paired *t* test on log-transformed data. [c]$n$ = number of tumours. For the group treated with aromatase inhibitors, 18 patients were treated, one patient having two tumours. [d]Patients on tamoxifen have been excluded.

**Table II** Changes in receptor concentration and tumour volume in large primary tumours according to response to systemic therapy

| *Response group* | *Treatment* | *Pre-treatment value*[a] | *Post-treatment value*[a] | *Difference* | *Significance* |
|---|---|---|---|---|---|
| *Oestrogen receptor concentration (fmol mg$^{-1}$ protein)* | | | | | |
| Regression ($n$ = 33)[c] | | | | | |
| Endocrine[b] (17) | | 102 | 127 | 1.26 ±2.96 | n.s. |
| Chemotherapy (13) | | 3 | 4 | 1.15 ±2.69 | n.s. |
| Endo[b] + Chemo (3) | | 43 | 34 | −1.26 ±2.89 | n.s. |
| No significant regression ($n$ = 23) | | | | | |
| Endocrine[b] (11) | | 79 | 70 | −1.14 ±3.08 | n.s. |
| Chemotherapy (4) | | 8 | 6 | −1.36 ±7.66 | n.s. |
| Endo[b] + Chemo (8) | | 33 | 24 | −1.38 ±2.63 | n.s. |
| *Clinical tumour volume (cm$^3$)*[d] | | | | | |
| Regression | all | 53.4 | 9.0 | −6.86 ±2.93 | $P$<0.001 |
| No significant regression | all | 34.7 | 28.4 | −1.25 ±1.36 | $P$<0.005 |

[a]Geometric means calculated after logarithmic transformation ± one standard deviation. [b]Patients on tamoxifen have been excluded ($n$ = 6). [c]$n$ = number of tumours, one patient having two tumours. [d]Tumour diameter was measured and response was classified as described previously (Anderson *et al.*, 1989). The results were converted to a tumour volume to give a better indication of tumour bulk, using the formula $4/3\pi r^3$, where $r$ = mean tumour radius.

As a control, the change in tumour volume for these two response groups was also examined. As expected, the group of patients showing significant regression, taken as a whole, exhibited a highly significant decrease in tumour volume. Although the remaining patients *individually* did not show a significant reduction in tumour volume, as a group they also exhibited a small decrease.

Examination of the relationship between changes in ER and change in tumour volume in individual patients (data not shown) equally did not reveal any consistent pattern.

### *Changes in ER concentration in relation to tumour morphology*

Although most treatments were, on average, without significant effect on ER concentration, in some individual patients there were large changes in tumour ER. In order to see if these related to tumour heterogeneity and sampling, the histological sections from 12 paired (pre- and post-treatment) tumour specimens were examined by the pathologist, in the absence of any knowledge of the ER concentration.

Of six paired tumour specimens showing a 'large' change in receptor concentration, four showed major differences in morphology between the pre- and post-treatment specimens. By contrast, none of the six paired specimens from patients showing little or no change in ER concentration exhibited any striking difference in histopathological appearance.

### Discussion

This study has demonstrated that, on average, tumour ER concentration is little changed by most forms of systemic therapy. Large changes in tumour ER concentration in individual patients were probably related to tumour heterogeneity (Hawkins *et al.*, 1977*a*; Van Netten, 1985; Senbanjo *et al.*, 1986). Patients on tamoxifen, however, did show a marked fall in receptor concentration during therapy; this was almost certainly due to interference by tamoxifen or its metabolites in the ligand-binding assay, as noted by Hull *et al.* (1983). In the present study, patients treated by medical or surgical oophorectomy showed only a slight, but insignificant rise in tumour ER concentration. In a large number of patients with fibroids, treated with the LHRH agonist, zoladex, however, a similar but *significant* rise in the concentration of ER in the uterine tissues has been observed (Lumsden *et al.*, 1989).

Previous studies in patients with breast cancer (Taylor *et al.*, 1982; Hamm & Allegra, 1988; Toma *et al.*, 1986) and in experimental animals (Vignon & Rochefort, 1976; Hawkins *et al.*, 1977*b*; Cho-Chung *et al.*, 1978) have shown a decrease in receptor concentration after endocrine manipulation or, as in the present study, no consistant change (Hull *et al.*, 1983; Mobbs *et al.*, 1987). The conflicting results in human breast cancer may derive from the inclusion of patients on tamoxifen (Taylor *et al.*, 1982), which causes a marked apparent reduction in ER concentration (this study and Hull *et al.*, 1983) or from the difficulties in comparing different tumour deposits (Taylor *et al.*, 1982; Hamm & Allegra, 1988).

In summary, ER concentration in breast tumours changed little after most common forms of systemic therapy, even in regressing tumours. Thus, in general, a marked change in ER concentration does not appear to be a component of the mechanism by which tumours are initially influenced by systemic therapy.

We are particularly grateful to Dr T.J. Anderson, Department of Pathology, for selecting the portions of tumour for assay, for carrying out the histopathological examination and helpful discussion. We thank the Cancer Research Campaign for support of Miss E.D.C. Anderson and Dr P.A. Levack (grant no. SP1256 to Professor Forrest). The receptor assays were performed by Miss A.L. Tesdale and Mr D. Carson, through the support of the Lothian Health Board. Miss K. Sangster and Mrs E. Killen kindly helped to check and collate the results.

### References

ANDERSON, E.D.C., FORREST, A.P.M., LEVACK, P.A., CHETTY, U & HAWKINS, R.A. (1989). Response to endocrine manipulation and oestrogen receptor concentration in large operable breast cancer. *Br. J. Cancer*, **60**, 223.

BRADFORD, M.M. (1976). A rapid and sensitive method for the quantitation of microgram quantities of protein utilizing the principle of protein-dye binding. *Anal. Biochem.*, **72**, 248.

CHO-CHUNG, Y.S., BODWIN, J.S. & CLAIR, T. (1978). Cyclic AMP-binding proteins. Inverse relationship with oestrogen receptors in hormone-dependent tumour regression. *Eur. J. Biochem.*, **86**, 51.

FORREST, A.P.M., LEVACK, P.A., CHETTY, U. & 4 others (1986). A human tumour model. *Lancet*, **ii**, 840.

HAMM, T.J. & ALLEGRA, J.C. (1988). Loss of hormonal responsiveness in cancer. In *Endocrine Management of Cancer. 1. Biological Bases*, Stoll, B.A. (ed.) p. 61. Karger: Basel.

HAWKINS, R.A. BLACK, R., STEELE, R.J.C., DIXON, J.M.J. & FORREST, A.P.M. (1981). Oestrogen receptor concentration in primary breast cancer and axillary node metastases. *Breast Cancer Res. Treat.*, **1**, 245.

HAWKINS, R.A., HILL, A. & FREEDMAN, B. (1975). A simple method for the determination of oestrogen receptor concentrations in breast tumours and other tissues. *Clin. Chim. Acta*, **64**, 203.

HAWKINS, R.A. HILL, A., FREEDMAN, B., GORE, S., ROBERTS, M.M & FORREST, A.P.M. (1977*a*). The reproducibility of measurements of oestrogen receptor concentration in breast cancer. *Br. J. Cancer*, **36**, 355.

HAWKINS, R.A., HILL, A., FREEDMAN, B. & 4 others (1977*b*). Oestrogen receptor activity and endocrine status in DMBA-induced rat mammary tumours. *Eur. J. Cancer*, **13**, 233.

HOEHN, J.L., PLOTKA, E.D. & DICKSON, K.B. (1979). Comparison of estrogen receptor levels in primary and regional metastatic carcinoma of the breast. *Ann. Surg.*, **190**, 69.

HULL, D.F., CLARK, G.M., OSBORNE, C.K., CHAMNESS, G.C., KNIGHT, W.A. & MCGUIRE, W.L. (1983). Multiple estrogen receptor assays in human breast cancer. *Cancer Res.*, **43**, 413.

LUMSDEN, M.A., WEST, C.P., HAWKINS, R.A., RUMGAY, L. & BAIRD, D.T. (1989). The binding of steroids of myometrium and leiomyomata (fibroids) in women treated with gonadotrophin-releasing hormone agonist (Zoladex, ICI, 118630). *J. Endocrinol.*, **121**, 389.

MOBBS, B.G., FISH, E.B., PRITCHARD, K.I., OLDFIELD, G. & HANNA, W.H. (1987). Estrogen and progestogen receptor content of primary and secondary breast carcinoma. *Eur. J. Cancer Clin. Oncol.*, **23**, 819.

SCATCHARD, G. (1949). The attraction of proteins for small molecules and ions. *Ann. NY Acad. Sci.*, **51**, 660.

SENBANJO, R.O., MILLER, W.R. & HAWKINS, R.A. (1986). Variations in steroid receptors and cyclic AMP binding proteins across human breast cancers: evidence for heterogeneity. *Br. J. Cancer*, **54**, 127.

TAYLOR, R.E., POWLES, T.J., HUMPHREYS, J. & 5 others (1982). Effects of endocrine therapy on steroid receptor content of breast cancer. *Br. J. Cancer*, **45**, 80.

TOMA, S., LECLERQ, G., HEUSON, J.C., LEONESSA, F. & PARIDENS, R. (1986). Estrogen receptor variations after systemic treatment. *Ann. NY Acad. Sci.*, **464**, 547.

VAN NETTEN, J.P., ALGARD, F.T., COY, P. & 6 others (1985). Heterogenous estrogen receptor levels detected via multiple microsamples from individual breast cancers. *Cancer*, **56**, 2019.

VIGNON, F. & ROCHEFORT, H. (1976). Regulation of estrogen receptors in ovarian-dependent rat mammary tumours. I. Effects of castration and prolactin. *Endocrinology*, **98**, 722.